# A long-distance inhibitory system regulates haustoria numbers in parasitic plants

**DOI:** 10.1073/pnas.2424557122

**Published:** 2025-02-18

**Authors:** Anna Kokla, Martina Leso, Jan Šimura, Cecilia Wärdig, Marina Hayashi, Naoshi Nishii, Yuichiro Tsuchiya, Karin Ljung, Charles W. Melnyk

**Affiliations:** ^a^Department of Plant Biology, Linnean Center for Plant Biology, Swedish University of Agricultural Sciences, Uppsala 756 51, Sweden; ^b^Department of Forest Genetics and Plant Physiology, Umeå Plant Science Centre, Swedish University of Agricultural Sciences, Umeå 90183, Sweden; ^c^Department of Biological Science, Graduate School of Science, Nagoya University, Nagoya 464-8601, Japan; ^d^Institute of Transformative Bio-Molecules, Nagoya University, Nagoya 464-8601, Japan

**Keywords:** haustoria, cytokinin signaling, parasitic plants

## Abstract

The ability of parasitic plants to withdraw nutrients from their hosts depends on the formation of an infective structure known as the haustorium. How parasites regulate their haustoria numbers is poorly understood, and here, we uncovered that existing haustoria in the facultative parasitic plants *Phtheirospermum japonicum* and *Parentucellia viscosa* suppressed the formation of new haustoria located on distant roots. Using *Phtheirospermum,* we found that this effect depended on the formation of mature haustoria and could be induced through the application of external nutrients. To understand the molecular basis of this root plasticity, we analyzed hormone response and found that existing infections upregulated cytokinin-responsive genes first at the haustoria and then more distantly in *Phtheirospermum* shoots. We observed that infections increased endogenous cytokinin levels in *Phtheirospermum* roots and shoots, and this increase appeared relevant since local treatments with exogenous cytokinins blocked the formation of both locally and distantly formed haustoria. In addition, local overexpression of a cytokinin-degrading enzyme in *Phtheirospermum* prevented this systemic interhaustoria repression and increased haustoria numbers locally. We propose that a long-distance signal produced by haustoria negatively regulates future haustoria, and in *Phtheirospermum*, such a signaling system is mediated by a local increase in cytokinin to regulate haustoria numbers and balance nutrient acquisition.

## Significance

Parasitic plants are globally important pathogens that withdraw nutrients from their hosts using infective structures known as haustoria. Despite the huge agricultural losses caused by parasitic plants, we know little about how infection is regulated. We found that in the parasitic plants *Phtheirospermum japonicum* and *Parentucellia viscosa*, existing haustoria prevented the formation of new haustoria located on distant roots. In *Phtheirospermum*, haustoria increased cytokinin levels and response locally. This cytokinin increase corresponded with the repression of haustoria both locally and systemically. By modifying cytokinin levels locally, we could enhance or inhibit long-distance repression. We propose that a negative feedback system exists in parasitic plants to control haustoria numbers in response to nutrient demands allowing parasites to regulate infection plasticity.

Plant parasitism has evolved at least 12 independent times resulting in thousands of different parasitic plant species that share a common feature: an infective structure known as the haustorium (1). Several parasitic plant species are important agricultural pests including *Striga* and *Cuscuta* that cause major economic losses every year (2, 3). These two species are obligate parasitic plants that depend entirely on their hosts for survival but many other species are facultative parasites that are independent of their hosts and instead parasitize under the right conditions (4). Many parasitic plants have important ecological roles, for example, they contribute to the spread of other species and biodiversity by parasitizing dominant species within their ecosystem (5). Using the haustorium, parasitic plants invade their hosts and form xylem connections, and some species also form phloem connections, to uptake water, nutrients, hormones, and RNAs (6–9).

Among the most studied parasitic plants is the facultative root parasite *Phtheirospermum japonicum*, an Orobanchaceae family member native to east Asia that infects a wide range of host species including *Arabidopsis thaliana*, tomato, maize, cowpea, and rice (7, 10). *Phtheirospermum* initiates prehaustoria formation in response to haustorium inducing factors (HIFs) released by hosts such as 2,6-dimethoxy-1,4-benzoquinone (DMBQ) (11–13). Following initiation, the prehaustorium attaches to the host root with the help of specialized root hairs (14), penetrates the host tissues using cell wall-modifying enzymes (15) and matures to form a haustorium with xylem connections known as the xylem bridge (10). In addition to HIFs, endogenously produced compounds such as auxin and ethylene are important for successful haustoria formation and xylem bridge development (10, 16). Negative regulators of haustoria formation are less well known but include exogenous nitrogen which, in *Phtheirospermum*, suppresses haustoria numbers via the upregulation of abscisic acid (17).

Plant hormones play important roles in the regulation and development of haustoria. One such hormone, cytokinin, is synthesized by ISOPENTENYLTRANSFERASE (IPT) and LONELY GUY (LOG) proteins, and these compounds can either act locally or move long distances where they are perceived by ARABIDOPSIS HISTIDINE KINASE (AHK) receptors to activate type B ARABIDOPSIS RESPONSE REGULATOR (ARR) transcription factors and cytokinin-degrading CYTOKININ OXIDASE (CKX) enzymes (18). Cytokinin levels increase at the site of *Phtheirospermum* infection and move from the parasite to the host to induce host root expansion (7, 19). Cytokinins also play important roles in other symbiotic relationships such as during parasitism by nematodes, when nematodes release cytokinins to activate cell division and form syncytium feeding sites (20). Similarly, cytokinins are essential for the formation of symbiotic structures called nodules that form between legumes and nitrogen-fixing bacteria (21). Such symbiotic relationships are often characterized by both positive and negative regulators that help balance the numbers of symbiotic structures to optimize resources use by the plant, a process which in legumes is known as autoregulation of nodulation (AON) (22).

Although the regulation of symbiosis is well known in legumes, it remains unknown whether parasitic plants regulate their haustorial numbers, and if so, which signals might control such a process. Here, we identified a system in *Phtheirospermum* whereby existing haustoria control the formation of new haustoria via a systemic repressive signal. Such a system was also present in another facultative root parasite, *Parentucellia viscosa*, an Orobanchaceae family member native to Europe. We investigated the increase in cytokinins caused by *Phtheirospermum* during infection and found that they served an inhibitory role for the formation of new haustoria found on both local and distant roots. We propose that cytokinin regulates haustoria formation as part of a long-distance repressive signal, thus allowing the parasite to control haustoria numbers and infection plasticity.

## Results

### A Systemic Signal Controls Haustoria Numbers.

**Fig. 1.**
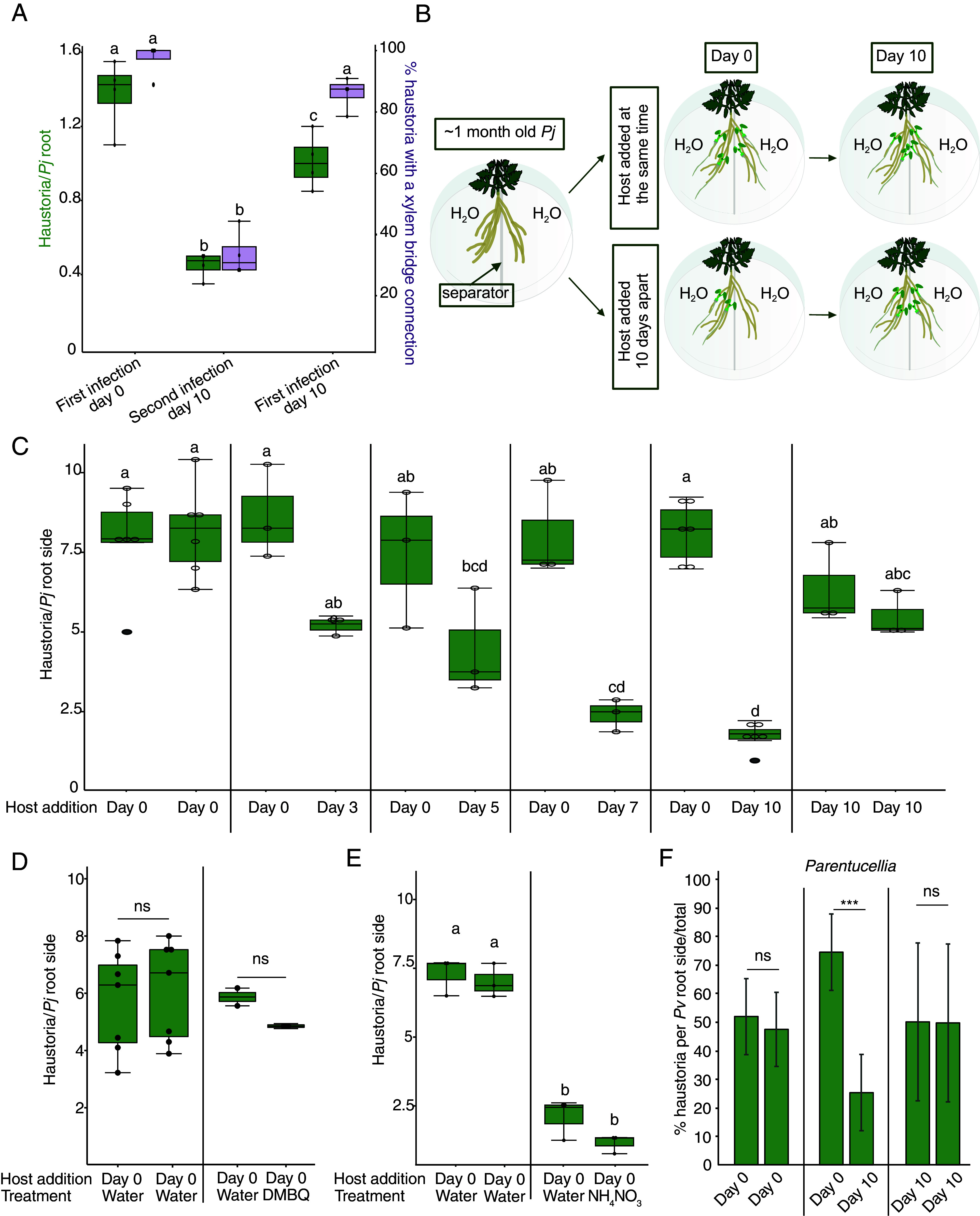
Haustoria numbers are controlled by systemic signaling. (*A*) Numbers of haustoria and percentage of haustoria with xylem bridges per *Phtheirospermum* with infection at day 0 followed by a second infection at day 10 on the same root. Control is first infection at day 10. The haustoria were evaluated 7 days after each infection. (n = 3 replicates). (*B*) Drawing of the split-root experimental setup. (*C*) Average number of haustoria per *Phtheirospermum* root side in a split-root setup on water agar, with host added on one side at 0 days postinfection (Day 0, dpi) and on the other side 3, 5, 7, or 10 days later, or on both sides at 0 or 10 dpi. (n = 3 to 6 replicates). (*D*) Average number of haustoria per *Phtheirospermum* root side in a split-root setup on water agar or 10 μM DMBQ, with host added on both sides at day 0. (n = 2 to 6 replicates). (*E*) Average number of haustoria per *Phtheirospermum* root side in a split-root setup on water agar or 10.3 mM NH_4_NO_3_, with host added on both sides at day 0. (n = 3 replicates). (*F*) Percentage of haustoria per *Parentucellia* root side in a split-root setup on water agar, with host *Arabidopsis* added on one side at 0 dpi (Day 0) and on the other side 10 days later, or on both sides at 0 or 10 dpi. (n = 4 to 7 plants, one-tailed Student’s *t* test, ***P < 0.001, ns = not significant). (*A* and *C*–*E*) Different letters represent *P* < 0.05, one-way ANOVA followed by Tukey’s HSD test.

Plants involved in nutrient-acquiring symbioses often regulate the extent of symbiosis (22, 23) but whether such regulation exists in plant parasitism is unknown. To investigate whether plant parasites control their number of nutrient-acquiring organs, the haustoria, we infected *Arabidopsis* with *Phtheirospermum* at day 0, followed by a second infection of a new *Arabidopsis* host added on the same *Phtheirospermum* root 10 days later (Fig. 1*A*). This second infection showed significantly fewer haustoria and reduced xylem bridge development compared to the first infection. To exclude age or starvation time effects, we performed first infections on plants at 10 days. These infections showed intermediate haustoria numbers but no difference in xylem bridge development compared to first infections at 0 days postinfection (dpi). To investigate whether such a phenomenon might operate over long distances, we developed a “split-root” system where *Phtheirospermum* roots were separated on two sides (Fig. 1*B*). When the host was added at the same time on both sides (Day 0-Day 0), both sides of the *Phtheirospermum* root system formed the same number of haustoria (Fig. 1*C*). We then added the hosts to one side at day 0 and waited 3, 5, 7, or 10 days before adding the hosts to the second side. While the Day 0 side formed the same number of haustoria as the Day 0-Day 0 control, the second side showed a progressive reduction in haustoria numbers (Fig. 1*C*). The age and starvation time control (Day 10-Day 10) had comparable numbers of haustoria to the Day 0-Day 0 plants (Fig. 1*C*). The haustorium inducing factor DMBQ causes prehaustoria to form in *Phtheirospermum* that do not mature to form xylem bridges (14), so we pretreated one side with DMBQ and found it did not inhibit the formation of haustoria on the distant side (Fig. 1*D*). In addition, removing the host added on the first side after 5 days of infection inhibited haustoria formation on the second side (*SI Appendix*, Fig. S1*A*). Together, these data suggested that mature haustoria were needed for systemic inhibition and that the inhibitory signal lasted for several days even if hosts were removed. Nutrients can regulate symbiosis, and in *Phtheirospermum*, exogenous nitrogen inhibits haustoria formation via ABA signaling (17). Applying 10.3 mM NH_4_NO_3_ to one side of the split-root setup inhibited haustoria both locally and systemically suggesting that nitrogen could function as part of a negative regulatory signal (Fig. 1*E*). However, ABA treatment inhibited haustoria formation only locally indicating ABA was not mobile or part of the systemic signaling process (*SI Appendix*, Fig. S1*B*), thus leaving the identity of the endogenous signal unresolved. To test whether systemic inhibition was found in other plant parasites, we performed split plate assays with *Parentucellia* and found that, similar to *Phtheirospermum*, existing haustoria suppressed the formation of new haustoria (Fig. 1*F*) indicating this regulatory system was not unique to *Phtheirospermum*.

### Infection Increases Systemic Cytokinin Response and Levels.

**Fig. 2.**
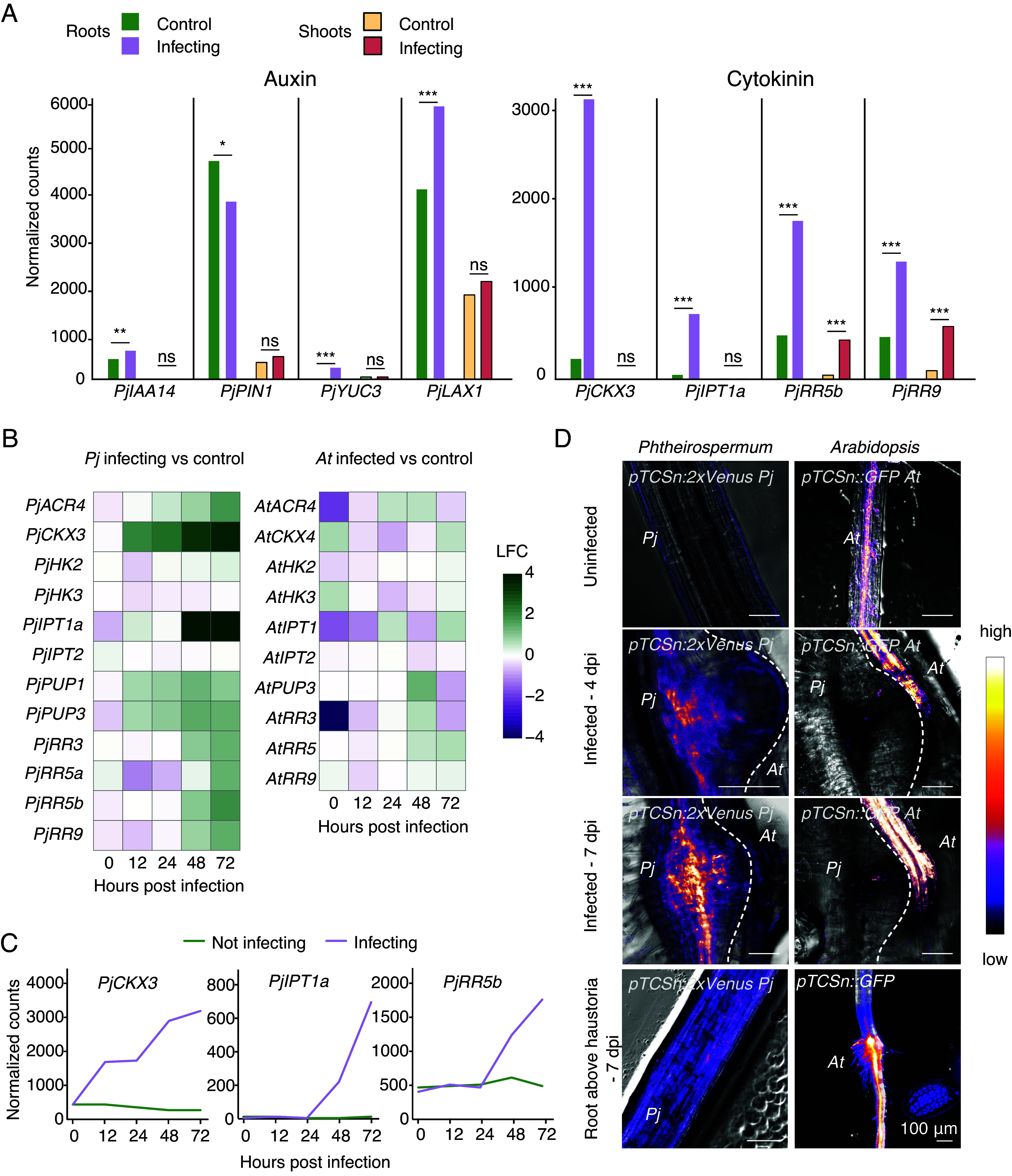
Infection induces systemic transcriptional changes in *Phtheirospermum*. (*A*) Normalized counts of auxin and cytokinin-related genes in *Phtheirospermum* infecting or control roots at 72 hours postinfection (hpi), and infecting or control shoots at 10 days postinfection (dpi). (n = 3 libraries, Wald test with Benjamini–Hochberg correction, *P < 0.05, **P < 0.01, ***P < 0.001, ns = not significant). (*B*) Heatmaps of cytokinin-related genes at 0, 12, 24, 48, or 72 hpi in water infect versus water control RNAseq libraries in *Phtheirospermum* and *Arabidopsis* roots. LFC = log2 fold change. (*C*) Normalized counts of *PjCKX3*, *PjIPT1a,* and *PjRR5b* in *Phtheirospermum* infecting or control roots at 0, 12, 24, 48, or 72 hpi. (*D*) Images of fluorescent *pTCSn* cytokinin reporters in *Arabidopsis* (*At*) or *Phtheirospermum* (*Pj*) for uninfected controls, 4 or 7 dpi haustoria and root above haustoria. (Scale bar, 100 μm).

**Fig. 3.**
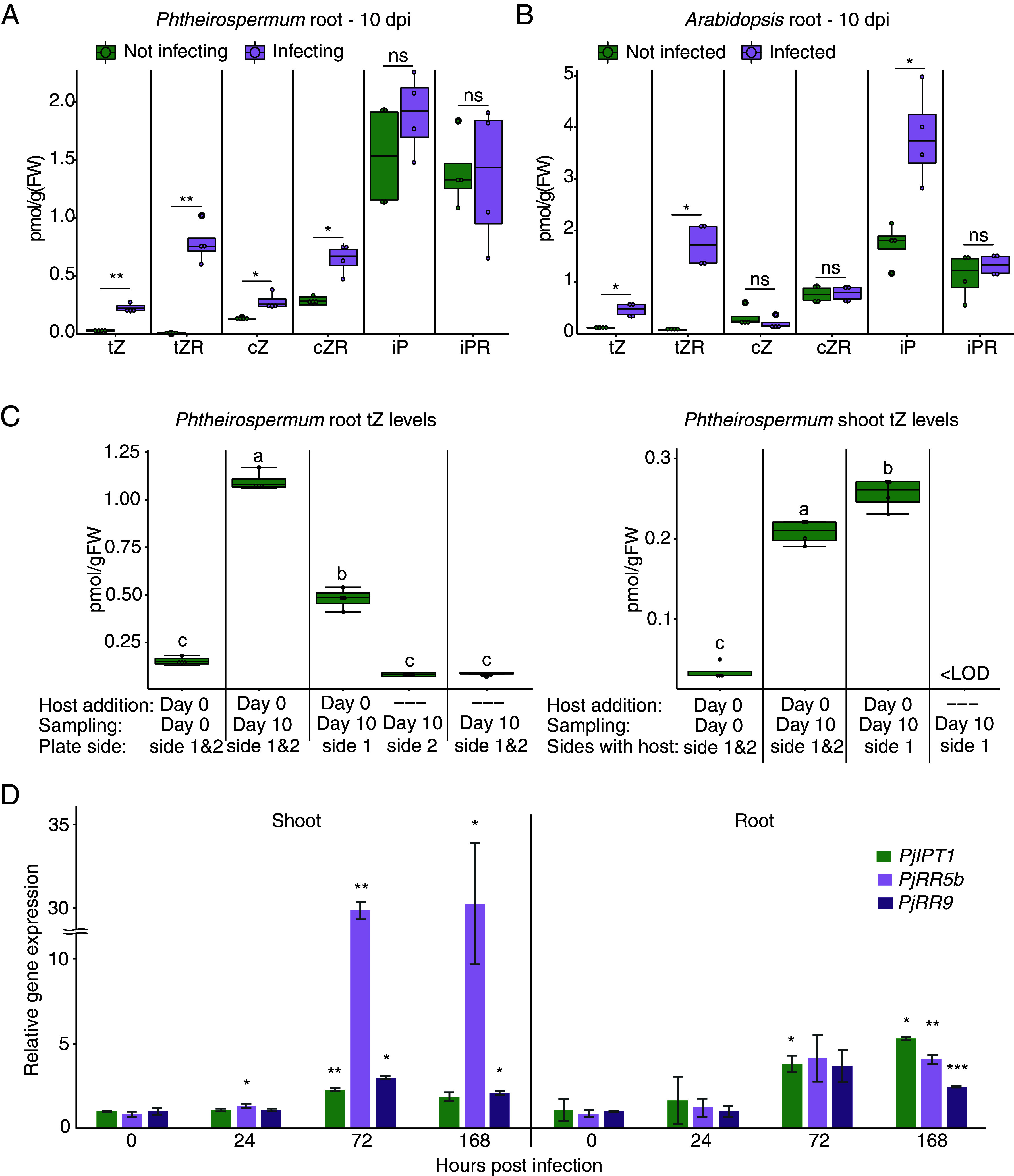
Cytokinin signaling increases systemically in *Phtheirospermum* after infection (*A*) Hormonal quantification of cytokinins in *Phtheirospermum* roots infecting or not infecting *Arabidopsis* at 10 days postinfection (dpi). (n = 4 replicates, Student *t* test with Benjamini–Hochberg correction, *P < 0.05, **P < 0.01, ns= not significant). (*B*) Hormonal quantification of cytokinins in *Arabidopsis* roots infected or not infected by *Phtheirospermum* at 10 dpi. (n = 4 replicates, Student *t* test with Benjamini–Hochberg correction, *P < 0.05, ns= not significant). (*C*) Quantification of tZ levels in *Phtheirospermum* roots and shoots in a split-root experimental setup. (n = 4 replicates, one-way ANOVA followed by Tukey’s HSD test). (*D*) qRT-PCR gene expression quantification of cytokinin-related genes in *Phtheirospermum* shoots and roots at 0, 24, 72, 168 hours postinfection (hpi), normalized to 0 hpi. (n = 2 replicates, Student’s *t* test, *P < 0.05, **P < 0.01, ***P < 0.001).

To understand how haustoria are systemically repressed in *Phtheirospermum*, we undertook a genome-wide RNAseq analysis in *Phtheirospermum* shoots at 10 dpi and found several hundred genes differentially expressed compared to control uninfected plants (*SI Appendix*, Fig. S1 *C*–*E*). These included genes related to DNA replication, signal transduction, cell wall modification, and response to biotic stimuli (*SI Appendix*, Fig. S1*E*). Given that hormones are important for haustoria formation (10, 24), we analyzed previously published transcriptomes of infecting roots at 72 h postinfection (hpi) (17) and our infecting shoot datasets for differentially expressed genes related to auxin, cytokinin, brassinosteroid, and gibberellic acid. Genes related to brassinosteroids [*BRI1-ASSOCIATED RECEPTOR KINASE1* (*PjBAK1*), *BR INSENSITIVE1* (*PjBRI1*), *BRASSINOSTEROID-INSENSITIVE2* (*PjBIN2*), and *BRASSINAZOLE RESISTANT1*(*PjBZR1*)] and gibberellic acid [*BETA HLH PROTEIN93* (*PjbHLH93*), *REPRESSOR OF GA* (*PjRGA1*), *GIBBERELLIN 20-OXIDASE1* (*PjGA20ox1*), and *GA INSENSITIVE DWARF1* (*PjGID1*)] did not show a clear trend of differential expression in either shoot or root. Auxin-related genes (*INDOLE-3 ACETIC ACID14* (*PjIAA14)*, *YUCCA3* (PjYUC3), *LIKE AUXIN RESISTANT1* (*PjLAX1*), and *PIN-FORMED1* (*PjPIN1*)) were differentially expressed only locally at the site of infection (Fig. 2*A* and *SI Appendix*, Fig. S2*A*). However, cytokinin-related genes *CYTOKININ OXIDASE3* (*PjCKX3*), *ISOPENTENYLTRANSFERASE1* (*PjIPT1a*), and *RESPONSE REGULATORs 5b* and *9* (*PjRR5b*, *PjRR9*) were all upregulated in infecting roots, and RRs were also upregulated in shoots of infecting plants (Fig. 2*A*). This upregulation of cytokinin response at the *Phtheirospermum* infection site occurred for many cytokinin-related genes, already by 12 hpi for genes like *PjCKX3* and the cytokinin transporters *PURINE PERMEASEs PjPUP1* and *PjPUP3* (Fig. 2 *B* and *C* and *SI Appendix*, Fig. S2*B*). In infected *Arabidopsis*, little cytokinin-related gene induction was observed by 72 hpi, perhaps in part due to the early sampling points before xylem bridge formation (Fig. 2*B*). Next, we confirmed the increase in cytokinin signaling during infection using the *pTCSn* cytokinin-responsive reporter (25). *pTCSn* was induced by four days after infection in *Phtheirospermum* and *Arabidopsis* at the haustorium site and in the root above haustoria (Fig. 2*D*). An increase in *pTCSn* signal was also observed in the hypocotyl vasculature of *Arabidopsis* infected at 7 dpi and in flower buds of infected plants at 30 dpi, but not in *Phtheirospermum* roots below the haustoria (*SI Appendix*, Fig. S2*C*). At 10 dpi, roots of infecting *Phtheirospermum* had significantly higher levels of the cytokinin species tZ, tZR, cZ, and cZR compared to noninfecting roots (Fig. 3*A*), while roots of infected *Arabidopsis* showed significantly higher levels of tZ, tZR, and iP compared to uninfected controls (Fig. 3*B*). We then measured the levels of cytokinin in roots and shoots of *Phtheirospermum* in the split-root setup. tZ and tZR levels increased in *Phtheirospermum* infecting roots 10 days after infection, but not in distant noninfecting roots (Fig. 3*C* and *SI Appendix*, Fig. S3*A*). tZ levels increased in shoots of infecting *Phtheirospermum*, either when one or both sides were infected, while tZR levels only slightly increased in shoots when just one side was infected (Fig. 3*C* and *SI Appendix*, Fig. S3*A*). Gene expression of the cytokinin-related genes *PjRR5* and *PjHK3* also increased in both roots and shoots when one side of the root system was infected (*SI Appendix*, Fig. S3*B*). The cytokinin biosynthesis homolog *PjIPT1* was upregulated in root RNAseq datasets by infection so we tested its expression by qPCR in shoots and found that it was not significantly increased at 7 dpi in infecting shoots, opposite to that of *PjRR5b* and *9* (Fig. 3*D*). Together, these results suggest an induction of cytokinin production and response at both the site of local infection and in shoot tissues, consistent with the movement of cytokinins from root to shoot in *Phtheirospermum*.

### Cytokinin Is a Local Inhibitor of Haustoria Development.

**Fig. 4.**
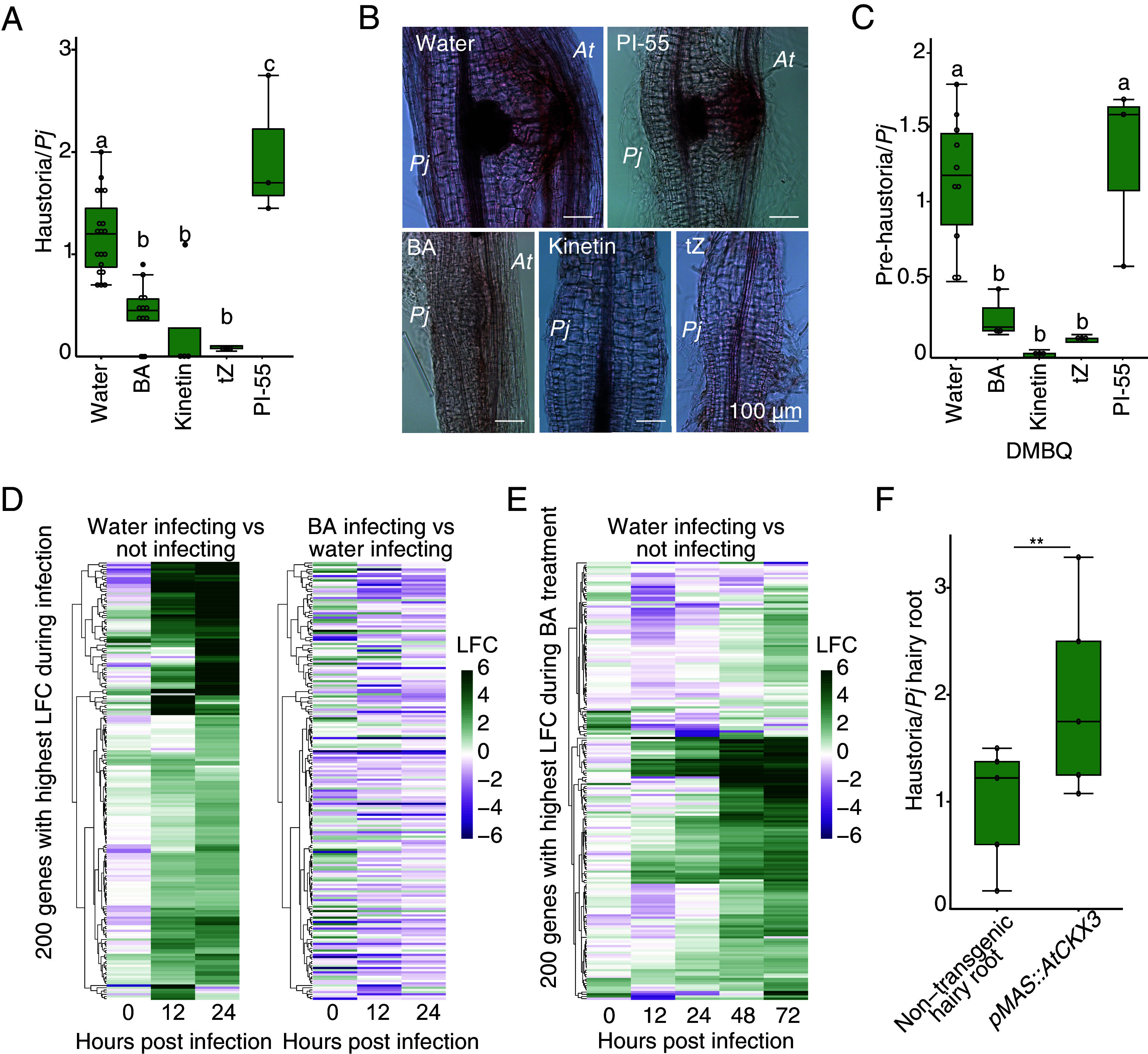
Cytokinin is a local inhibitor of haustoria development. (*A*) Average number of haustoria per *Phtheirospermum* seedling in in vitro infection assays with 80 nM BA, 100 μM kinetin, 1 μM tZ, 1 μM PI-55, or water control at 7 days postinfection (dpi). (n = 3 to 21 replicates, one-way ANOVA followed by Tukey’s HSD test). (*B*) Brightfield images of Safranin-O stained *Phtheirospermum* (*Pj*) haustoria at 7 dpi with chemical treatments. (Scale bar, 100 μm). (*C*) Average number of prehaustoria per *Phtheirospermum* seedling in in vitro haustorium induction assays with 10 μM DMBQ and water, 80 nM BA, 100 μM kinetin, 1 μM tZ, or 1 μM PI-55. (n = 3 to 10 replicates, one-way ANOVA followed by Tukey’s HSD test). (*D*) Heatmap of 200 genes with the highest log2 fold change (LFC) during haustoria formation shown over three time points in water infecting versus not infecting and BA infecting versus water infecting *Phtheirospermum* root RNAseq libraries. (*E*) Heatmap of the 200 genes with the highest LFC after BA treatment shown over five time points in water infecting versus not infecting *Phtheirospermum* roots RNAseq libraries. (*F*) Haustoria per *Phtheirospermum* root in infection assay using *Phtheirospermum* with nontransgenic hairy roots or hairy roots overexpressing *AtCKX3*. (n = 5 replicates, Student *t* test, **P < 0.01).

An increase in cytokinin levels during plant parasitism is associated with host root growth (7), but the role of the cytokinin increase in the parasite is unknown. We performed exogenous cytokinin treatments during infection assays. Application of 80 nM BA, 100 μM kinetin, and 1 μM trans-zeatin significantly reduced haustoria induction (Fig. 4 *A* and *B*), while inhibiting cytokinin signaling by applying the cytokinin antagonist PI-55 (1 μM) increased haustoria numbers (Fig. 4 *A* and *B*). To test whether this cytokinin-mediated haustoria inhibition required a host, we induced prehaustoria using DMBQ with no host and found exogenous cytokinins significantly reduced the number of DMBQ-induced prehaustoria, while PI-55 had no significant effect (Fig. 4*C* and *SI Appendix*, Fig. S3*C*). We infected the *Arabidopsis* cytokinin-related mutants *cre1ahk3*, *ckx3ckx5*, *p35S:CKX1*, *arr1,12*, *arrx8*, *ahp6-3*, and *ipt161* and observed no significant difference in haustoria numbers compared to wild type Col-0 control (*SI Appendix*, Fig. S3*D*). We then analyzed a transcriptome dataset where *Phtheirospermum* infecting *Arabidopsis* was treated with BA and haustorium tissues were harvested at 0, 12, and 24 hpi (17). More than 1,000 genes were differentially expressed in the BA infecting samples compared to the water infecting samples and in BA infecting versus BA control samples (*SI Appendix*, Fig. S4*A*). Analyses of BA infect samples compared to water infect samples identified three different patterns of coexpression (*SI Appendix*, Fig. S4*B*). Cluster 1, whose gene expression decreased at 12 hpi, had an overrepresentation of genes related to protein processing, sucrose transport, and transcription regulation (*SI Appendix*, Fig. S4*C*). Cluster 2, whose gene expression peaked at 12 hpi, had an overrepresentation of genes related to transcriptional and translational processes (*SI Appendix*, Fig. S4*C*). Cluster 3, whose gene expression peaked at 24 hpi, had an overrepresentation of genes related to oxidoreduction processes (*SI Appendix*, Fig. S4*C*). Many of the genes that were highly upregulated during parasitism were downregulated by BA treatment, consistent with BA repressing a haustoria-inducing program (Fig. 4*D*). Furthermore, genes that were BA responsive in *Phtheirospermum* and *Arabidopsis* were also upregulated during infection, consistent with cytokinin response increasing as the haustoria matured (Fig. 4*E* and *SI Appendix*, Fig. S4*E*). To further investigate the role of cytokinin, we overexpressed the cytokinin-degrading *Arabidopsis* CKX3 enzyme in *Phtheirospermum* hairy roots (*SI Appendix*, Fig. S4*F*). Transformed hairy roots formed significantly more haustoria on Col-0 hosts compared to nontransgenic hairy roots (Fig. 4*F*), consistent with parasite-derived cytokinins being important for inhibiting haustoria formation.

### Cytokinin Mediates a Systemic Haustoria-Repressing Signal.

**Fig. 5.**
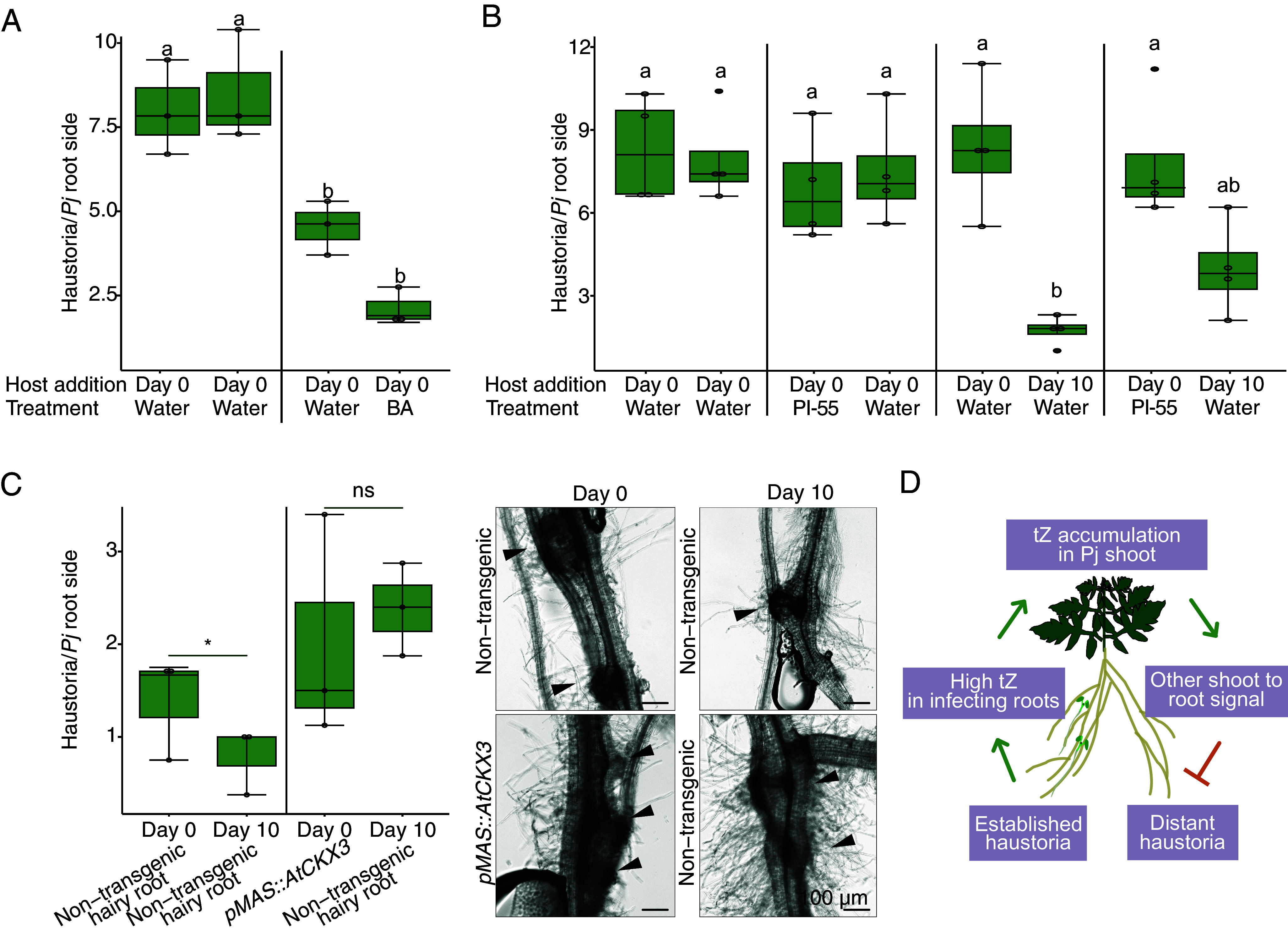
Cytokinin mediates the systemic regulation of haustoria numbers. (*A*) Average number of haustoria per *Phtheirospermum* in a split-root setup on water agar or 80 nM BA, with host added on both sides at 0 days postinfection (dpi) (n = 3 replicates, one-way ANOVA followed by Tukey’s HSD test). (*B*) Average number of haustoria per *Phtheirospermum* in a split-root setup on water agar or 1 μM PI-55, with host added on both sides at 0 dpi or one side at 0 dpi and 10 days later on the other side. (n = 4 replicates, one-way ANOVA followed by Tukey’s HSD test). (*C*) Average number of haustoria per *Phtheirospermum* in a split-root setup with nontransgenic hairy roots or hairy roots overexpressing *AtCKX3*, and representative brightfield images. Arrowheads point at haustoria. (Scale bar, 100 μm). (n = 3 replicates, Student’s *t* test, *P < 0.05 ns = not significant). (*D*) Proposed model of cytokinin-mediated haustoria regulation in *Phtheirospermum*.

We next investigated the role of cytokinin in long-distance haustoria repression. When 80 nM BA was applied to one side of the split-root setup, both treated and distant root sides significantly reduced haustoria numbers compared to the control (Fig. 5*A*), suggesting a systemic repressive role for cytokinin. We then investigated whether the local increase in cytokinin biosynthesis and signaling following infection is needed for systemic repression. We treated one side of the split-root setup with 1 μM PI-55 and found PI-55 treatment had no significant effect on haustoria numbers at day 0. However, the distant side infected at 10 days had slightly reduced haustoria numbers but not significantly different to the PI-55 treated side infected at 0 days (Fig. 5*B*), showing local application of PI-55 partially inhibited the repressive signal in distant roots. Finally, we sought to endogenously modify cytokinin signaling in *Phtheirospermum* by infecting transgenic hairy roots overexpressing *AtCKX3* at day 0, followed by infection of a nontransgenic hairy root on the same plant at day 10. While the control nontransgenic plants showed fewer haustoria on the day 10 root side (Fig. 5*C*), the transformed plants showed no significant difference in the numbers of haustoria between the day 0 and day 10 infections (Fig. 5*C*). Taken together, these data indicated that local cytokinin production or response in infecting roots was needed to initiate systemic signaling that regulates future haustoria formation in *Phtheirospermum* (Fig. 5*D*).

## Discussion

Plants involved in nutrient-acquiring symbioses with mycorrhiza and nitrogen-fixing bacteria regulate their extent of symbiosis (23) and here, we uncover in the facultative parasitic plants *P. japonicum* and *P. viscosa* a regulatory system whereby existing haustoria control the formation of new haustoria. This system required cytokinin whose increase during parasitism is known from work in *Phtheirospermum*, *Cuscuta,* and *Santalum* yet, to date, this increase has only been associated with a developmental response in the host (7, 26, 27). Such parasite-derived cytokinins promote tissue expansion in the host but notably not in the parasite (7), suggesting different roles for these cytokinins in parasite and host. By using a combination of exogenous treatment assays and transgenic approaches, we demonstrated here that parasite-derived cytokinins act as haustoria-repressing factors that control local and long-distance haustoria numbers. The identity of the mobile signals mediating systemic repression remains unknown given that our exogenous application assays and transgenic studies focused on the infecting roots. However, trans-zeatin species were highly increased in the shoot despite little or late upregulation of cytokinin biosynthesis genes in the shoot, and cytokinin transporters were also upregulated in infected roots (Fig. 3 *A* and *C* and *SI Appendix*, Figs. S2*B* and S3*A*). Thus, we propose that root-produced cytokinins likely act as a root-to-shoot signaling component associated with systemic suppression, consistent with the known mobility of cytokinins from root to shoot through the xylem in *Arabidopsis* (28). We did not observe an increase in cytokinins in distant roots (Fig. 3*C* and *SI Appendix*, Fig. S3*A*), indicating the shoot-to-root haustoria-repressing factor remains unknown.

Notably, in both *Striga hermonthica* and *Phelipanche ramosa*, cytokinins act as haustoria-inducing factors (29, 30). These findings contrast with our own data and suggest that cytokinins have different roles in the obligate parasites *Striga* and *Phelipanche* compared to the facultative parasite *Phtheirospermum*, perhaps due to their lifestyle or physiology. *Striga* and *Phelipanche* form haustoria by differentiating the ends of primary root tips, and there, haustoria regulation may occur via a primary root formation or differentiation pathway. In contrast, *Phtheirospermum* form lateral haustoria where divisions in the epidermis, cortex, stele, and endodermis are relevant (31). Lateral root initiation is inhibited by cytokinin (32), whereas cytokinins promote primary root differentiation (33) providing a possible explanation for differences between *Striga* and *Phtheirospermum*. Lateral root numbers are also controlled by nutrient-based feedback regulation in plants and a similar situation may occur with haustoria in parasitic plants. Providing low or moderate nitrogen levels promotes lateral root formation, whereas high nitrogen levels suppress lateral root formation (34). In facultative parasitic plants, exposure to a nutrient source like a host might promote haustoria formation, while an abundance of haustoria and hosts might suppress more haustoria, forming parallels to lateral root formation during nitrogen foraging and demonstrating the importance of root plasticity.

Successful symbioses are often characterized by a combination of negative and positive regulators. In legumes, autoregulation of nodules integrates environmental inputs such as nitrogen with a negative regulation pathway, CLE-SUNN, and a positive regulation pathway, CEP-CRA2, to optimize nodule numbers (35). In the parasitic plant *Phtheirospermum*, several positive signals have been identified, including haustoria-inducing factors such as DMBQ, and hormones such as auxin that promotes haustoria initiation, and ethylene that promotes host invasion (10, 12, 16). Recent work in *Phtheirospermum* has found that auxin-related compounds move from shoots to root to promote haustoria maturation (36) and also identified CLE peptides as positive regulators of haustoria formation (37). However, in *Phtheirospermum* and *Striga*, only nitrogen has been identified as a local haustoria suppressing factor (17). Here, we show that cytokinin is also repressive and extend this previous observation to show that nutrients can also act systemically to regulate haustoria numbers. Such environmental information as well as negative and positive regulators could form parts of a long-distance system regulating haustoria numbers. Given the presumed need to balance resource expenditure with resource acquisition during parasitism, we expect there to be additional negative regulators of haustoria formation in *Phtheirospermum*, *Striga,* and other parasitic plants. Discovering these compounds and understanding the prevalence of systemic haustoria regulation and how such a system functions in parasitic plants should be a priority. In addition, deploying and introducing such negative regulators in the host could provide durable resistance to parasites.

## Materials and Methods

### Plant Materials and Growth Conditions.

*P. japonicum* (Thunb.) Kanitz ecotype Okayama seeds were described previously (38). *P. viscosa* seeds used in this study were harvested at Nagoya, Japan, in 2023. *Arabidopsis* ecotype Columbia (Col-0) was used as *Arabidopsis* wild-type. *Arabidopsis cre1-12ahk3-3*, *ckx3-1ckx5-2, 35S::CKX1, arr1,12*, *arrx8*, *ahp6-3*, *ipt161*, and *pTCSn::GFP* were published previously (25, 39–45). For in vitro germination, *Phtheirospermum* and *Arabidopsis* seeds were surface sterilized with 70% (v/v) EtOH for 20 min followed by 95% (v/v) EtOH for 5 min. The seeds were then placed on ½MS medium (0.8% (w/v) plant agar and 1% (w/v) sucrose, pH 5.8). After overnight stratification in the dark and 4 °C, the plants were placed to long day conditions (16-h light:8-h dark and light levels 100 μmol m^−2^ s^−1^) and 25 °C. For *Parentucellia*, ~500 seeds were surface sterilized in a 1.5 mL tube by rinsing with 20% solution of commercial bleach for three times, followed by ethanol three times and distilled water for three times. The tube was covered with tin foil and rotated at 16 °C for one week. The seeds were plated on 0.7% agarose/water plate and germinated for two weeks at 16 °C in 12 h light:12 h dark condition.

### In Vitro Infection Assays with *Phtheirospermum*.

The in vitro infection assays were performed following a published protocol (17). Four- to five-day-old *Phtheirospermum* seedlings were transferred for three days to nutrient-free 0.8% (w/v) agar medium or 0.8% (w/v) agar medium supplemented by hormone treatments: 0.08 μM 6-Benzylaminopurine (BA), 1 μM PI-55, 1 μM trans-zeatin, or 100 μM kinetin. Five-day-old *Arabidopsis* seedlings were aligned next to and roots place in contact with the pretreated *Phtheirospermum* roots for infection assays. Haustoria numbers were measured at seven days postinfection using a Zeiss Axioscope A1 microscope. For prehaustorium assays, seedlings after the three-day starvation/hormone treatment were transferred to 0.8% (w/v) agar medium containing 10 μM DMBQ (Sigma-Aldrich) with or without hormonal treatment. Prehaustoria were counted at seven days.

### Split-Root Infection Assays with *Phtheirospermum* and *Parentucellia*.

One-month-old *Phtheirospermum* were transferred on Gosselin™ polystyrene round petri plates (split-plate) (Fisher Scientific) with nutrient-free 0.8% (w/v) agar medium on both sides or 0.8% (w/v) agar medium supplemented by 10.3 mM NH_4_NO_3_, 0.08 μM BA, 1 μM ABA, or 1 μM PI-55 on one side. The root system was separated in the 2 sides of the split plate, so they are not in contact with each other. Four days later, two 7-day-old *Arabidopsis* (Col-0) hosts were added in alignment with the roots of *Phtheirospermum* on either both sides or one of them. In the nonhost root sides, the hosts were added 10 days later (10 dpi). The measurements of haustoria numbers were taken at 7 days after host addition. For split root assays with *Parentucellia*, seedlings were incubated after germination in the same growth condition as for the *Phtheirospermum* split root assay until the root length reached to 2 to 3 cm (~1.5 months). The assay was then performed as described for *Phtheirospermum*.

### Cloning of *pTCSn:2xVenus-NLS* for Expression in *Phtheirospermum*.

The cloning was based on the Greengate method following the standard protocols (46). The primers used for GreenGate cloning are listed in *SI Appendix*, Table S1. Digestion and ligation reactions were performed using the BsaI-HFv2 (NEB #R3733) and T4 DNA Ligase (NEB #M0202) enzymes, respectively. The *pTCSn* promoter fragment was cloned from a previously published vector (7) using the CloneAmp™ HiFi PCR Premix (TakaraBio) and inserted into the entry vector *pGGA000* (Addgene plasmid #48856) to create the *pGGA-pTCSn* vector. The ligated plasmid was amplified in *E. coli* DH5α and confirmed by Sanger sequencing. The binary vector assembly was performed using *pGGA-pTCSn, pGGB003* (Addgene plasmid #48821*), pGGC-2xVenus-NLS* (15), *pGGD002* (Addgene plasmid #48834), *pGGE-tMAS* (17), *pGGF-DsRed* (17), and *pGGZ001* (Addgene plasmid #48868). The final plasmid was cotransformed in electrocompetent *Agrobacterium rhizogenes* AR1193 with the pSoup plasmid (Addgene plasmid #165419), and the bacteria cultured in LB broth with 50 ug/ml spectinomycin and 50 ug/ml rifampicin.

### *Phtheirospermum* Transformation.

Transformation was performed as described previously (47). Briefly, five-day-old *Phtheirospermum* seedlings were sonicated for 10 to 15 seconds followed by vacuum infiltration for 5 min with suspension of *Agrobacterium rhizogenes* strain AR1193 carrying the *Arabidopsis CKX3* gene overexpressing construct, *pMAS::AtCKX3:tMAS*, previously described (7), or *pTCSn::2xVenus-NLS:tMAS*. The seedlings were then transferred on cocultivation media (Gamborg’s B5 medium, 0.8% agar, 1% sucrose, 450 μM acetosyringone) first at 20 °C for 2 days in dark conditions, then at 25 °C under long-day conditions for ~1 month with 300 μg/ml cefotaxime. Transgenic hairy roots were infected as described above. Both transgenic and nontransgenic hairy roots showed reduced levels of infection compared to nonhairy roots, likely due to transformation conditions slightly reducing plant vigor.

### Histological Staining, Microscopy, and Confocal Imaging.

For visualization of xylem bridges, dissected *Phtheirospermum* roots were fixed in ethanol-acetic acid and stained with Safranin-O solution (0.1%) followed by clearing with chloral hydrate for two to three days before microscopic observation with a Zeiss Axioscope A1 microscope as described previously (14). A Leica M205 FA stereo microscope was used with RFP filter for the selection of transformed *Phtheirospermum* hairy roots. In Arabidopsis root-hypocotyl junction, hypocotyl and flowers, the *TCSn* fluorescent reporter was visualized using a Leica M205 FA stereo microscope with GFP filter. *Arabidopsis* and *Phtheirospermum TCSn* roots were visualized on a Zeiss LSM780 laser scanning confocal microscope with 514 nm excitation, 2.5% laser power, 518 to 624 nm emission.

### Sample Preparation for RNAseq.

Sample preparation for the root infection time course in control (water) conditions and following BA treatment was previously described (17). For the preparation of the *Phtheirospermum* shoot sequencing, four *Phtheirospermum* seedlings of four- to five-day-old per sample were transferred to nutrient-free 0.8% (w/v) agar medium for 3 days prior to infection with *Arabidopsis* Col-0. As a control, four *Phtheirospermum* seedlings remained without the *Arabidopsis* host. The shoots of the *Phtheirospermum* seedlings were collected at 10 dpi. These experiments were replicated three times. RNA extraction was performed using the ROTI®Prep RNA MINI (Roth) kit following the manufacturer’s instructions. The isolation of mRNA and library preparation were performed using NEBNext® Poly(A) mRNA Magnetic Isolation Module (#E7490), NEBNext® Ultra™ RNA Library Prep Kit for Illumina® (# E7530L), NEBNext® Multiplex Oligos for Illumina® (#E7600) following the manufacturer’s instructions. The libraries were then sequenced using paired-end sequencing with an Illumina NovaSeq 6000.

### Bioinformatic Analysis.

Bioinformatic analysis of the water and BA sequencing data have been previously described (17). The same method was followed for the shoot sequencing data. Briefly, the adapter and low-quality sequences were removed using the fastp software with default parameters (48). The quality-filtered reads were mapped to the *Phtheirospermum* genome (16) using STAR (49) and the read count was calculated using FeatureCounts (50). The differential expression analysis was performed using Deseq2 (51) (Dataset S1). The gene expression clustering was performed using the Mfuzz software (52). Custom annotations of the *Phtheirospermum* predicted proteins (16) were estimated using InterProScan (53) and used for the gene ontology analysis that was performed using the topGO software (54). Cytokinin-related genes from *Arabidopsis* were blasted against the *Phtheirospermum* genome (16) using the tBLASTp and tBLASTn algorithms; these genes can be found in Dataset S2. The 200 genes with highest expression were identified by selecting the genes with LFC>1.5 in the *Phtheirospermum* water infecting vs not infecting RNAseq libraries for the 12 and 24 hpi time points, *Phtheirospermum* BA vs DMSO not infecting RNAseq libraries for the 0 hpi time point, and *Arabidopsis* BA vs DMSO not infected RNAseq libraries for the 24 hpi time point (Dataset S2).

### qRT-PCR.

For the split root setup, the root sides or shoots of three plants were collected for each replicate. For the infection time course, the roots or shoots of 5 infecting or control *Phtheirospermum* seedlings were harvested at 0, 24, 72, and 168 hours postinfection and frozen in liquid nitrogen. For the *AtCKX3* transformed hairy roots, four or five fluorescent or nonfluorescent (control) hairy roots were collected for each replicate. RNA extraction was performed using the ROTI®Prep RNA MINI (Roth) kit following the manufacturer’s instructions. cDNA synthesis was performed using the Maxima First Strand cDNA Synthesis Kit for RT-qPCR (Thermo Scientific™) following the manufacturer’s instructions. *PjPP2A* (10) was used as an internal control. qPCR was performed with SYBR-Green master mix (Applied Biosystems™). The relative expression was calculated using the Pfaffl method (55). The primers used for this experiment are listed in *SI Appendix*, Table S1.

### Hormonal Quantification.

For hormonal quantifications of roots in *Phtheirospermum* and *Arabidopsis*, *Phtheirospermum* seedlings were grown for five days before transferring to water agar for three days. *Arabidopsis* Col-0 was then placed next to the *Phtheirospermum* seedlings and left for 10 days. *Phtheirospermum* with no host were used as a control. Four to five *Phtheirospermum* or *Arabidopsis* whole seedlings were then harvested per sample with care taken to separate samples under a microscope to minimize sample mixing. For quantification on the split plate setup, ~ 1 month *Phtheirospermum* seedlings were placed on nutrient-free 0.8% (w/v) agar medium or 0.8% (w/v) agar medium on petri dishes with a plastic separator in the middle (split-plate) for seven days. *Arabidopsis* Col-0 was placed next to the *Phtheirospermum* roots on both or one of the split roots sides and left for 10 days. *Phtheirospermum* at 0 dpi were used as control. The root sides of three *Phtheirospermum* plants were collected for each sample at 0 dpi or 10 dpi, with care taken to separate host and parasite under a microscope to minimize sample mixing. The samples were crushed to powder using liquid nitrogen with mortar and pestle. Samples were extracted, purified, and analyzed according to a previously published method (56). Briefly, approx. 20 mg of frozen material per sample was homogenized and extracted in 1 mL of ice-cold 50% aqueous acetonitrile (v/v) with the mixture of ^13^C- or deuterium-labeled internal standards using a bead mill (27 hz, 10 min, 4 °C; MixerMill, Retsch GmbH, Haan, Germany) and sonicator (3 min, 4 °C; Ultrasonic bath P 310 H, Elma, Germany). The samples were then centrifuged (14,000 RPM, 15 min, 4 °C) and the supernatant was purified according to the following procedure. A solid-phase extraction column Oasis HLB (30 mg 1 cc, Waters Inc., Milford, MA) was conditioned with 1 ml of 100% methanol and 1 ml of deionized water (Milli-Q, Merck Millipore, Burlington, MA) followed by sample loading on the SPE column. The flow-through and elution fractions were collected in 1 ml 30% aqueous acetonitrile (v/v). The samples were then dried using speed vac (SpeedVac SPD111V, Thermo Scientific, Waltham, MA) and dissolved in 40 µL of 30% acetonitrile (v/v) in insert-equipped vials. Mass spectrometry analysis for the detection of targeted compounds was performed by an UHPLC–ESI–MS/MS system comprising a 1290 Infinity Binary LC System coupled to a 6490 Triple Quad LC/MS System with Jet Stream and Dual Ion Funnel technologies (Agilent Technologies, Santa Clara, CA). The quantification was carried out in Agilent MassHunter Workstation Software Quantitative (Agilent Technologies, Santa Clara, CA). Hormonal quantification values are provided in Dataset S3.

## Supplementary Material

Appendix 01 (PDF)

Dataset S01 (XLSX)

Dataset S02 (XLSX)

Dataset S03 (XLSX)

## Data Availability

RNA Sequence data are available at the Gene Expression Omnibus (http://www.ncbi.nlm.nih.gov/geo/) under accession numbers GSE177484 (57) and GSE253722 (58). Sequence data of the *Phtheirospermum* genes studied in this article are available in GenBank (http://www.ncbi.nlm.nih.gov/genbank/) under the accession numbers provided in *SI Appendix*, Table S2. All study data are included in the article and/or supporting information. Previously published data were used for this work (59).
