# Supplementary material for: A long-distance inhibitory system regulates haustoria numbers in parasitic plants
Source: Proc Natl Acad Sci U S A. 2025 Feb 18;122(8):e2424557122. doi: 10.1073/pnas.2424557122 (PMC11874510; doi:10.1073/pnas.2424557122)
Supplement: Supplementary file 1 — Appendix 01 (PDF) [file pnas.2424557122.sapp.pdf]

PNAS

Supporting Information for

A long-distance inhibitory system regulates haustoria numbers in parasitic plants

Anna Kokla*, Martina Leso*, Jan Simura, Cecilia Wärdig, Marina Hayashi, Naoshi Nishii, Yuichiro Tsuchiya, Karin Ljung, Charles W. Melnyk

Charles W. Melnyk
Email: charles.melnyk@slu.se

This PDF file includes:

Figures S1 to S4
Tables S1 to S2
Legends for Datasets S1 to S3

Other supporting materials for this manuscript include the following:

Datasets S1 to S3

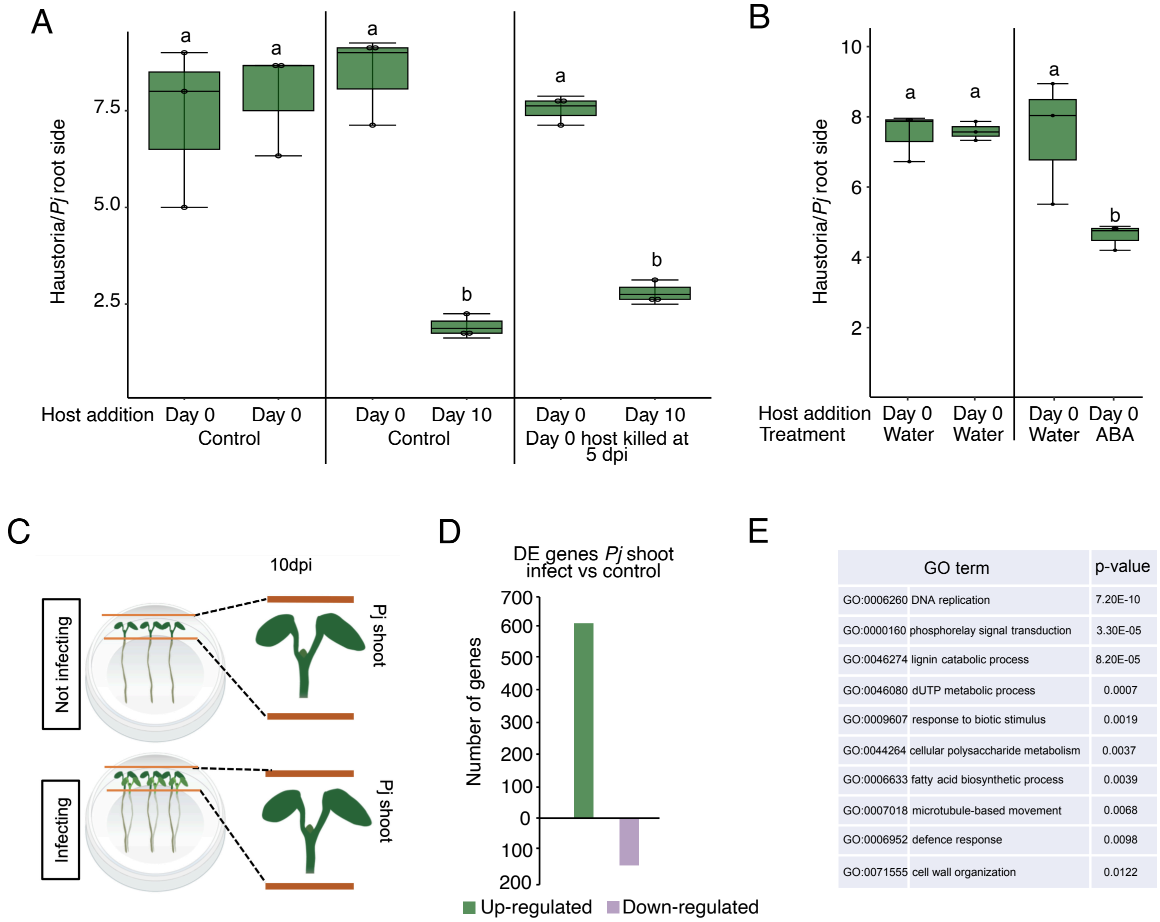

GO term		p-value
GO:0006260	DNA replication	7.20E-10
GO:0000160	phosphorelay signal transduction	3.30E-05
GO:0046274	lignin catabolic process	8.20E-05
GO:0046080	dUTP metabolic process	0.0007
GO:0009607	response to biotic stimulus	0.0019
GO:0044264	cellular polysaccharide metabolism	0.0037
GO:0006633	fatty acid biosynthetic process	0.0039
GO:0007018	microtubule-based movement	0.0068
GO:0006952	defence response	0.0098
GO:0071555	cell wall organization	0.0122

Fig. S1. ABA does not regulate haustoria numbers systemically

A) Average number of haustoria per *Phtheirospermum* in a split-root setup on water agar with host added on both sides at 0 days post infection (dpi) (day 0), or on one side at 0 dpi and other side at 10 dpi. In control samples, hosts were left alive throughout infection, or the host added at day 0 was killed at 5 dpi, before adding the host on the second side (n = 3 replicates, one-way ANOVA followed by Tukey's HSD test) **B)** Average number of haustoria per *Phtheirospermum* root side in a split-root setup on water agar or 1 µM ABA with host added on both sides at 0 dpi. (n = 3 replicates, one-way ANOVA followed by Tukey's HSD test) **C)** Drawings showing the experimental setup for the *Phtheirospermum* shoot sequencing. **D)** Number of genes differentially expressed between control and infecting *Phtheirospermum* shoots at 10 dpi. **E)** Gene ontology analysis for the differentially expressed genes between control and infecting *Phtheirospermum* shoots at 10 dpi (n = 3 libraries, Wald test with Benjamini-Hochberg correction, p<0.05).

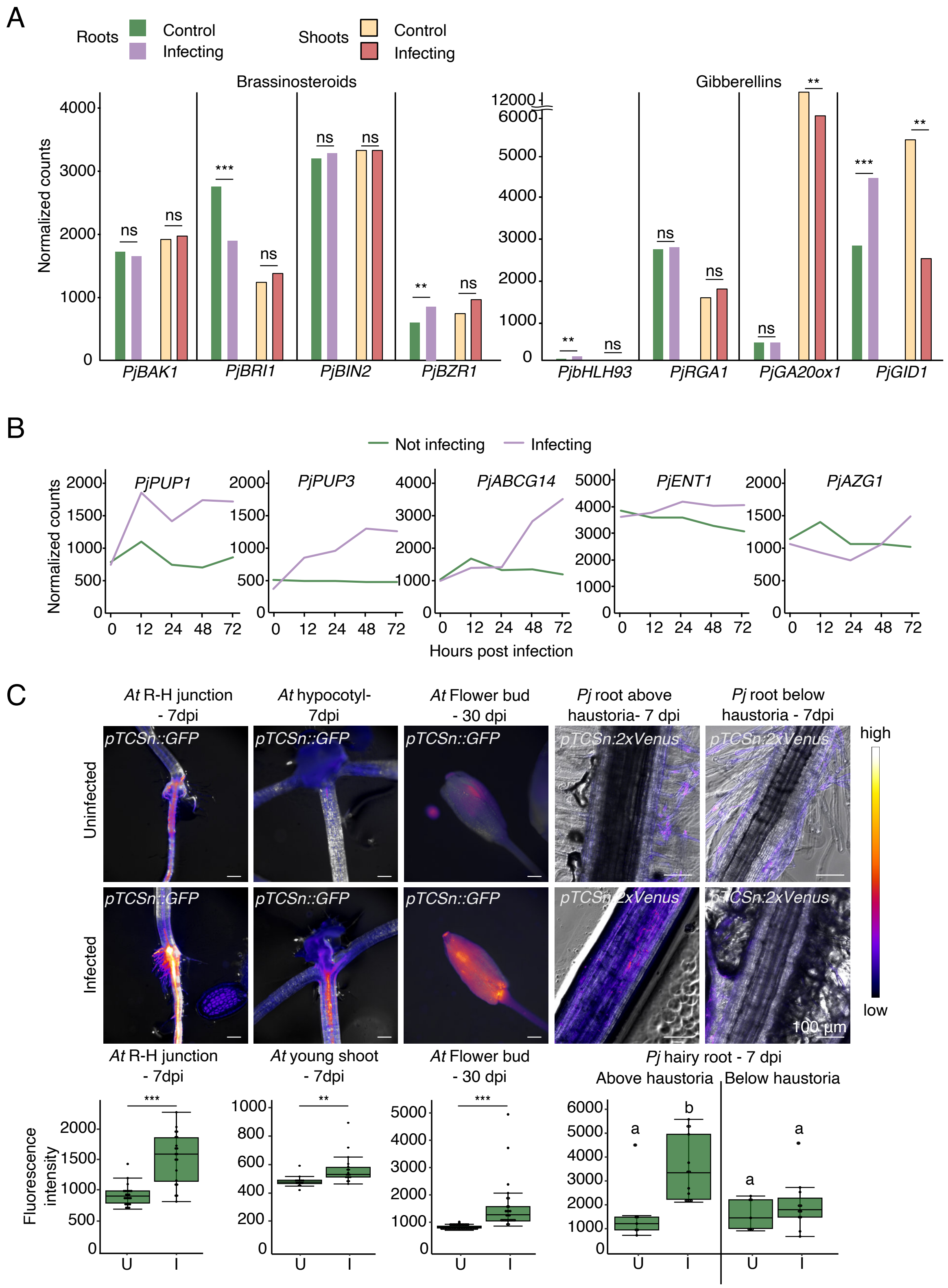

A
Roots
Control
Infecting
Shoots
Control
Infecting
Brassinosteroids
Gibberellins
Normalized counts
PjBAK1
PjBRI1
PjBIN2
PjBZR1
PjbHLH93
PjRGA1
PjGA20ox1
PjGID1
B
Not infecting
Infecting
PjPUP1
PjPUP3
PjABCG14
PjENT1
PjAZG1
Hours post infection
C
At R-H junction - 7dpi
At hypocotyl- 7dpi
At Flower bud - 30 dpi
Pj root above haustoria- 7 dpi
Pj root below haustoria - 7dpi
Uninfected
Infected
pTCSn::GFP
pTCSn:2xVenus
high
low
100 µm
At young shoot - 7dpi
Pj hairy root - 7 dpi
Above haustoria
Below haustoria
Fluorescence intensity
U
I

Fig. S2. Brassinosteroid and gibberellin signalling are not systemic in *Phtheirospermum* following infection

A) Normalized counts of brassinosteroid and gibberellin-related genes in *Phtheirospermum* infecting or control roots at 72 hours post infection (hpi), and infecting or control shoots at 10 days post infection (dpi). (n = 3 libraries, Wald test with Benjamini-Hochberg correction, ** p<0.01, *** p<0.001, ns = not significant) **B)** Normalized counts of *PjPUP1*, *PjPUP3*, *PjABCG14*, *PjENT1* and *PjAZG1* in *Phtheirospermum* infecting or control roots at 0, 12, 24, 48 or 72 hpi. **C)** Images and quantifications of fluorescent *pTCSn* cytokinin reporters for 7 dpi *Arabidopsis* root-hypocotyl (R-H) junctions and shoots, 1 month post infection *Arabidopsis* flower buds and 7 dpi *Phtheirospermum* hairy roots above (upper root) and below haustorium site (lower root). Scale bars 100 μm. *At* = *Arabidopsis, Pj = Phtheirospermum*, U = uninfected, I = infected. (for *Arabidopsis*: n = 20-41 images, ** p<0.01, *** p<0.001, Student t-test; for *Phtheirospermum*: n = 7-13 images, one-way ANOVA followed by Tukey's HSD test).

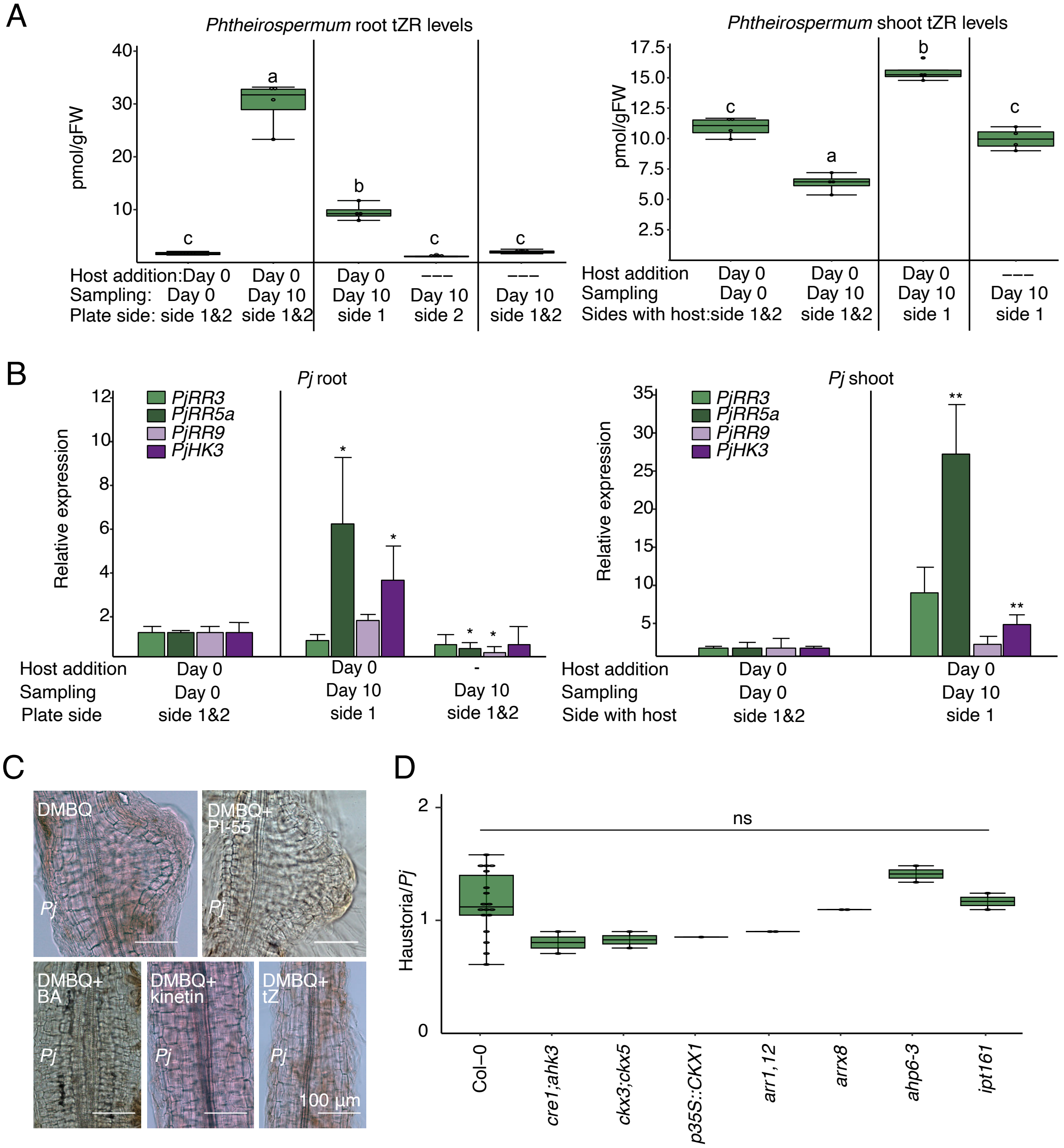

Fig. S3. Host cytokinin response does not influence haustoria induction

A) Quantification of tZR levels in *Phtheirospermum* roots and shoots in a split-root experimental setup. (n = 4 replicates, one-way ANOVA followed by Tukey's HSD test) **B)** qRT-PCR gene expression quantification of cytokinin-related genes in *Phtheirospermum* roots and shoots in a split-root setup. (n = 3 replicates, Student's t-test, * p<0.05, ** p<0.01). **C)** Brightfield images of *Phtheirospermum* (Pj) pre-haustoria at 7 days post infection (dpi) treated with DMBQ, cytokinins or PI-55. Scale bars 100 µm. **D)** Average number of haustoria at 7 dpi per *Phtheirospermum* seedling infecting *Arabidopsis cre1;ahk3*, *ckx3;ckx5*, *p35S::CKX1, arr1,12, arrx8, ahp6-3* and *ipt161* mutants, or Col-0 control. (n = 2-18 replicates, one-way ANOVA followed by Tukey's HSD test, ns = not significant)

A

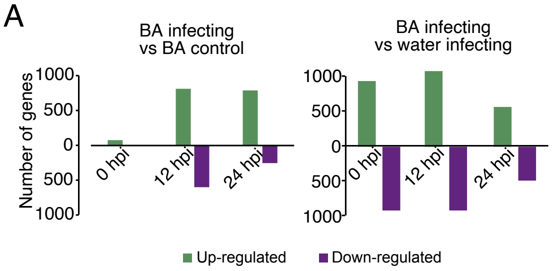

B

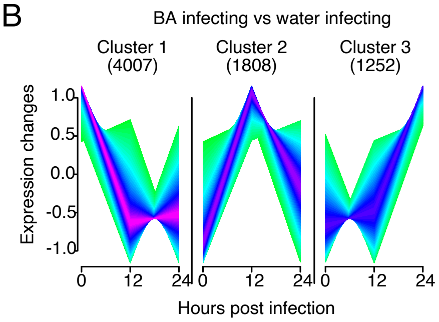

C

Cluster 1 (BA infecting vs water infecting)			Cluster 2 (BA infecting vs water infecting)			Cluster3 (BA infecting vs water infecting)		
GO term		p-value	GO term		p-value	GO term		p-value
GO:0006468	protein phosphorylation	1.20E-09	GO:0006260	DNA replication	3.30E-20	GO:0055114	oxidation-reduction process	0.003
GO:0016567	protein ubiquitination	0.00024	GO:0000105	histidine biosynthetic process	3.60E-05	GO:0006783	heme biosynthetic process	0.0041
GO:0005992	trehalose biosynthesis	0.002	GO:0006302	double-strand break repair	6.80E-05	GO:0015074	DNA integration	0.006
GO:1903506	regulation transcription	0.00312	GO:0009451	RNA modification	0.00012	GO:0006508	proteolysis	0.0085
GO:0015770	sucrose transport	0.00873	GO:0016072	rRNA metabolic process	0.00031	GO:0006450	regulation of translational fidelity	0.0443
GO:0006680	glucosylceramide catabolism	0.01807	GO:0022613	ribonucleoprotein biogenesis	0.00038	GO:0009229	thiamine diphosphate biosynthesis	0.0443
GO:0048268	clathrin coat assembly	0.03064	GO:0006457	protein folding	0.00079	GO:0032780	negative regulation of ATPase activity	0.0443
GO:0016573	histone acetylation	0.03494	GO:0009089	lysine biosynthetic process	0.00082			
GO:0023052	signaling	0.03505	GO:0006310	DNA recombination	0.00097			
GO:0000288	mRNA catabolism	0.041	GO:0006418	tRNA aminoacylation	0.00107			
GO:0006665	sphingolipid metabolism	0.04925	GO:0006207	de novo' pyrimidine biosynthesis	0.00333			
			GO:0032259	methylation	0.00472			

E

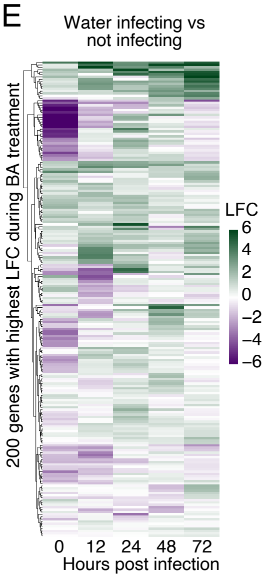

D

BA infecting vs BA control up-regulated								
0 hpi		p-value	12 hpi		p-value	24 hpi		p-value
GO:0009690	cytokinin metabolic process	3.30E-08	GO:000969	cytokinin metabolic process	1.90E-06	GO:0009690	cytokinin metabolic process	5.00E-06
GO:0006855	drug transmembrane transport	2.20E-07	GO:0006096	glycolytic process	4.00E-05	GO:0042744	hydrogen peroxide catabolic process	5.50E-06
GO:0000160	phosphorelay signal transduction system	1.50E-06	GO:0006108	malate metabolic process	0.00012	GO:0071555	cell wall organization	7.30E-05
GO:0009664	plant-type cell wall organization	0.00012	GO:0000160	phosphorelay signal transduction system	0.00015	GO:0055114	oxidation-reduction process	0.00012
GO:0042744	hydrogen peroxide catabolic process	0.00013	GO:0055114	oxidation-reduction process	0.00021	GO:0006979	response to oxidative stress	0.00018
GO:0006979	response to oxidative stress	0.00029	GO:0006006	glucose metabolic process	0.00022	GO:0045492	xylan biosynthetic process	0.00024
GO:0015770	sucrose transport	0.00628	GO:0051187	cofactor catabolic process	0.00044	GO:0008272	sulfate transport	0.00219
GO:0005986	sucrose biosynthetic process	0.03312	GO:0009607	response to biotic stimulus	0.00074	GO:0006869	lipid transport	0.0023
GO:0006006	glucose metabolic process	0.0025	GO:0006855	drug transmembrane transport	0.00099	GO:0042546	cell wall biogenesis	0.00484
GO:0009688	abscisic acid biosynthetic process	0.03312	GO:0042737	drug catabolic process	0.00099	GO:0010105	negative regulation of ethylene-activate...	0.04928
BA infecting vs BA ccontrol down-regulated								
0 hpi		p-value	12 hpi		p-value	24 hpi		p-value
GO:0006857	oligopeptide transport	0.00058	GO:0046274	lignin catabolic process	1.00E-05	GO:0007018	microtubule-based movement	4.10E-07
GO:0070588	calcium ion transmembrane transport	0.00214	GO:2001141	regulation of RNA biosynthetic process	1.50E-05	GO:0055085	transmembrane transport	0.00099
GO:0048544	recognition of pollen	0.00308	GO:0048544	recognition of pollen	0.00049	GO:0006857	oligopeptide transport	0.00469
GO:0009607	response to biotic stimulus	0.00365	GO:0055114	oxidation-reduction process	0.00061	GO:0030001	metal ion transport	0.0218
GO:0055114	oxidation-reduction process	0.00494	GO:0070588	calcium ion transmembrane transport	0.00203	GO:0000165	MAPK cascade	0.0296
GO:0006952	defense response	0.00659	GO:0009607	response to biotic stimulus	0.00337	GO:0005992	trehalose biosynthetic process	0.03229
GO:0008283	cell proliferation	0.00896	GO:0006950	response to stress	0.00409	GO:0048544	recognition of pollen	0.0331
GO:0042744	hydrogen peroxide catabolism	0.00949	GO:0006020	inositol metabolic process	0.00529	GO:0055114	oxidation-reduction process	0.04856
GO:0000154	rRNA modification	0.01317	GO:0000165	MAPK cascade	0.01128			
GO:0006809	nitric oxide biosynthetic process	0.03092	GO:0006809	nitric oxide biosynthetic process	0.03035			

F

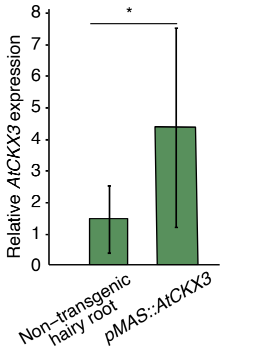

Fig. S4. Exogenous cytokinin treatment induces gene expression changes in *Phtheirospermum*

A) Number of differentially expressed genes over three time points in the BA infecting versus BA control and BA infecting versus water infecting RNA-seq libraries in *Phtheirospermum*. **B)** Clustering of differentially expressed (DE) genes in BA infecting versus water infecting RNA-seq datasets of *Phtheirospermum* infecting *Arabidopsis* over three time points based on their co-expression patterns. The number in parenthesis is the number of genes in each cluster. **C)** Gene ontology analysis for the DE genes assigned to each co-expression cluster for the BA infecting versus water infecting RNA-seq datasets (n = 3 libraries, Wald test with Benjamini-Hochberg

correction, p<0.05). **D)** Gene ontology analysis for the up or down regulated genes for the BA infecting versus BA control RNA-seq datasets (n = 3 libraries, Wald test with Benjamini-Hochberg correction, p<0.05). **E)** Heatmap of the 200 genes with the highest log2 fold change (LFC) after BA treatment shown over five time points in water infecting versus not infecting *Arabidopsis* roots RNAseq libraries. **F)** qRT-PCR gene expression quantification of *AtCKX3* in *Phtheirospermum* transgenic or non-transgenic hairy roots. (n = 5-6 replicates, Student's t-test, * p<0.05).

Table S1. List of primers used

Name	Forward	Reverse	Purpose
PjPP2A	GGGGTCTTTCACCCTCACTC	CATGCGGAACCTCCTGTGTA	qPCR
PjIPT1a	GAACGAGTACCTGGGGAAGC	CCGCCGTACCTCTCGAAATA	qPCR
PjRR3	GATTGCTCAAAATCACTTCTTGC	GAAACTGCAAAGCCCTAATCC	qPCR
PjRR5a	CGGTTAAGCTCGCAGACG	TTTTTCTCCTCGCCTCTCAA	qPCR
PjRR5b	CCTTTTGTTTCTCCCAAAATTC	TTCCAGGCATACAATAGTCTGTAATAA	qPCR
PjRR9	CGGAGAATATTCCGTCGAGA	GGCTTTAGGAAAAACTCTTGAGC	qPCR
PjHK3	CTCACAGCTCCGTTCAGGTT	CCTTTCGGCAGGGGTTGTAT	qPCR
AtCKX3	TCGAACCGGAGAGCTAAACC	CCGATCATTCCACTTGTTGCG	qPCR
pTCSn	tttGGTCTCaACCTGCTTGACTAGTCAAAGATCT	tttGGTCTCaTGTTGTTATATCTCCTTGGA	GreenGate cloning

Table S2. Accession numbers of *P. japonicum and A. thaliana* genes shown in figures.

Gene Name	Gene ID	Genbank accession	Locus tag	Protein accession
AtCKX3	AT5G56970	NM_001345226	AT5G56970	NP_001331716.1
PjACR4	Pjv1_00008943	BMAC01000167.1	PHJA_000984100	GFP88404.1
PjHK2	Pjv1_00008425	BMAC01000095.1	PHJA_000631900	GFP84881.1
PjHK3	Pjv1_00008929	BMAC01000167	PHJA_000982700	GFP88390
PjIPT1a	Pjv1_00009372	BMAC01000091.1	PHJA_000610000	GFP84661.1
PjIPT2	Pjv1_00021215	BMAC01000728.1	PHJA_002355700	GFQ02118.1
PjPUP1	Pjv1_00014382	BMAC01000175.1	PHJA_001033200	GFP88895.1
PjPUP3	Pjv1_00003989	BMAC01000035.1	PHJA_000331100	GFP81878.1
PjRR3	Pjv1_00020598	BMAC01000424	PHJA_001758700	GFP96146
PjRR5a	Pjv1_00020695	BMAC01000499	PHJA_001912700	GFP97686
PjRR5b	Pjv1_00002654	BMAC01000024.1	PHJA_000225800	GFP80825.1
PjRR9	Pjv1_00026921	BMAC01000994	PHJA_002638600	GFQ04945
PjABCG14	Pjv1_00003147	BMAC01000031.1	PHJA_000296000	GFP81527.1
PjENT1	Pjv1_00025562	BMAC01001133.1	PHJA_002737300	GFQ05933.1
PjAZG1	Pjv1_00025639	BMAC01001045.1	PHJA_002676900	GFQ05328.1
PjIAA14	Pjv1_00021591	BMAC01000581.1	PHJA_002101700	GFP99576.1
PjPIN1	Pjv1_00008680	BMAC01000122.1	PHJA_000768100	GFP86243.1
PjYUC3	Pjv1_00016159	BMAC01000425.1	PHJA_001762600	GFP96185.1
PjLAX1	Pjv1_00016802	BMAC01000347.1	PHJA_001582300	GFP94379.1
PjCKX3	Pjv1_00001077	BMAC01000016.1	PHJA_000167100	GFP80237.1
PjBAK1	Pjv1_00018983	BMAC01000540.1	PHJA_002011100	GFP98672.1
PjBRI1	Pjv1_00005476	BMAC01000060.1	PHJA_000468700	GFP83253.1
PjBIN2	Pjv1_00016899	BMAC01000252.1	PHJA_001313900	GFP91699.1
PjBZR1	Pjv1_00017214	BMAC01000287.1	PHJA_001415000	GFP92708.1
PjbHLH93	Pjv1_00008838	BMAC01000195.1	PHJA_001101800	GFP89581.1
PjRGA1	Pjv1_00006222	BMAC01000065.1	PHJA_000496400	GFP83530.1
PjGA20ox1	Pjv1_00009950	BMAC01000151.1	PHJA_000914200	GFP87705.1
PjGID1	Pjv1_00023589	BMAC01000741.1	PHJA_002378400	GFQ02344.1

Dataset S1 (separate file). Deseq2 results of *P. japonicum* shoot sequencing

Dataset S2 (separate file). Genes included in heatmaps

Dataset S3 (separate file). Cytokinin hormonal quantification values
